# Supplementary material for: Chromothripsis during telomere crisis is independent of NHEJ, and consistent with a replicative origin
Source: Genome Res. 2019 May;29(5):737–49. doi: 10.1101/gr.240705.118 (PMC6499312; doi:10.1101/gr.240705.118)
Supplement: Supplemental Material [file supp_gr.240705.118_Supplemental_file_1.zip › contigs/annotated_contigs/DB40/contig.2.DB40_length_631_mean_cov_10.6973058637.docx]

**DB40_length_631_mean_cov_10.6973058637**

```
GAGTGAGGGAATAGCATTCTCCAACATTGCTGTCTCTGCATATGTCTTTGTTACAAGGCAATGGCAGACTGCTACAGAGTGTAATGGAA
 >chr12:127280223-127280410 + E=9e-101
TGGTAAAGCTAAAATGGGTCATTTCCTAAAAACAGTGACATCAATCTGATTCTTCAAAGCATAGAGGTACTGAAGAACAAATAGGGAAC

CATGTGGG|C|CTTGCCCTTCCTGGTTGTTGGTGGAATTCAGTTCCTTGTGATTGTAGGATCAGGGTTCCTATTTGCTGTTGTTTGCTG
         >chr12:127280934-127281382 + E=1e-250
TAAACTTTAAACTGAGGACCACTCCTGGTGTCCAGAGGACACCTGCACTCATGGGATCATGGTCTTGTCCCTCTATTTTCAAAGCCAGC

AGTGTCAGAGGTCAAGGTCTCACCTTCCAACCTCCCTTTCTCCTTGTAAGGACTCTTGTGGTGTGTTGGGCCCACCCAGACAACCCAGG

GGCATCTTCCCATTTCAATATCAACTAATTAGAAATCTGAATTCCATCTGAAAACATAATTGTCTTTTGCTTTGTAAGGTAACATATTC

ATAGATTCTGGGGATTAGAACCTGGATGTCTTTGGAGGACCATTATTCTAACACACTTACCTAAACTGCCTGTGATACGGAAGCTGGAC

AGTGTATCTG
```
